# Alzheimer’s disease drug-development pipeline: few candidates, frequent failures

**DOI:** 10.1186/alzrt269

**Published:** 2014-07-03

**Authors:** Jeffrey L Cummings, Travis Morstorf, Kate Zhong

**Affiliations:** 1 Cleveland Clinic Lou Ruvo Center for Brain Health, 888 West Bonneville Avenue, Las Vegas, Nevada 89106, USA; 2 Touro University Nevada College of Osteopathic Medicine, Henderson, Nevada, USA

## Abstract

**Introduction:**

Alzheimer’s disease (AD) is increasing in frequency as the global population ages. Five drugs are approved for treatment of AD, including four cholinesterase inhibitors and an *N*-methyl-D-aspartate (NMDA)-receptor antagonist. We have an urgent need to find new therapies for AD.

**Methods:**

We examined Clinicaltrials.gov, a public website that records ongoing clinical trials. We examined the decade of 2002 to 2012, to better understand AD-drug development. We reviewed trials by sponsor, sites, drug mechanism of action, duration, number of patients required, and rate of success in terms of advancement from one phase to the next. We also reviewed the current AD therapy pipeline.

**Results:**

During the 2002 to 2012 observation period, 413 AD trials were performed: 124 Phase 1 trials, 206 Phase 2 trials, and 83 Phase 3 trials. Seventy-eight percent were sponsored by pharmaceutical companies. The United States of America (U.S.) remains the single world region with the greatest number of trials; cumulatively, more non-U.S. than U.S. trials are performed. The largest number of registered trials addressed symptomatic agents aimed at improving cognition (36.6%), followed by trials of disease-modifying small molecules (35.1%) and trials of disease-modifying immunotherapies (18%). The mean length of trials increases from Phase 2 to Phase 3, and the number of participants in trials increases between Phase 2 and Phase 3. Trials of disease-modifying agents are larger and longer than those for symptomatic agents. A very high attrition rate was found, with an overall success rate during the 2002 to 2012 period of 0.4% (99.6% failure).

**Conclusions:**

The Clinicaltrials.gov database demonstrates that relatively few clinical trials are undertaken for AD therapeutics, considering the magnitude of the problem. The success rate for advancing from one phase to another is low, and the number of compounds progressing to regulatory review is among the lowest found in any therapeutic area. The AD drug-development ecosystem requires support.

## Introduction

Alzheimer’s disease (AD) is becoming increasingly common as the global population ages. It is estimated that currently 44 million victims of AD dementia exist in the world and that this will grow to more than 100 million cases by 2050 [1,2]. We urgently need to identify drugs that prevent, delay the onset, slow the progression, or improve the symptoms of AD.

Drug development for AD has proven to be very difficult. Five drugs are approved for the treatment of AD including four cholinesterase inhibitors (tacrine, donepezil, rivastigmine, galantamine) and an *N*-methyl-D-aspartate (NMDA) receptor AD antagonist (memantine) [3,4]. No new treatments have been approved for AD since 2003. Tacrine was approved by the US Food and Drug Administration (FDA) in 1993, donepezil in 1996, rivastigmine in 1998, galantamine in 2001, and memantine in 2003 (made available in the United States in 2004). Many failures in AD drug development have occurred, with both small molecules and immunotherapies failing to show a drug/placebo difference or having unacceptable toxicity [5-8].

To understand better the process of drug development for AD, we conducted an analysis of clinicaltrials.gov, a government website that serves the mandate to record all ongoing clinical trials. We analyzed both trial activity and, where possible, unique compound progress through the AD pipeline. We examined all trials since 2002 and conducted a separate analysis of currently ongoing trials and currently active compounds. Our goal was to examine historic trends to help understand why AD treatment development efforts so often fail and to provide insight into AD drug development.

## Methods

Clinicaltrials.gov is a public website that records ongoing clinical trials of all diseases. The database began in 2000 [9]. In 2005, the International Committee of Medical Journal Editors (ICMJE) began to require trial registration in a public database as a condition of publication [10]. This greatly increased the number of registrants on clinicaltrials.gov. Beginning in 2007, the FDA Amendments Act required registration of all clinical trials of drugs and devices subject to FDA regulation [11]. Registration is required no later than 21 days after enrollment of the first participant. Clinicaltrials.gov provides reliable data on clinical trials starting from this 2007 date.

Clinicaltrials.gov provides comprehensive information in text form about trials. The description includes trial name, sponsor, name of agent, phase of trial, inclusion and exclusion criteria, primary and secondary outcomes, number of participants, duration of trial, and location of trial sites.

We used the advanced-search mechanisms of clinicaltrials.gov to construct a comprehensive database that included the year the trial was registered, phase of trial (1,2,3), funder, drug name, clinical trials identification number, study type, status of the trial (active, not recruiting, recruiting, completed, terminated), date last updated, study start date, study estimated end date, number of participants to be enrolled, length of treatment intervention, location of study (U.S. only, non-U.S. only, both U.S. and non-U.S.), Mini Mental State Examination (MMSE) score inclusion criteria, AD condition (cognitively normal persons in prevention trials, prodromal AD, mild cognitive impairment (MCI), AD dementia), sponsor, allocation (randomized or not), end-point classification (safety, efficacy, and so on), intervention model (single group, parallel group, cross-over), masking (double-blind, open label), and official title of trial.

Funders were analyzed as industry, National Institutes of Health (NIH), NIH plus industry, other federal agencies (such as Department of Veterans Affairs), and all others (including academic medical centers). Mechanism of action was also recorded for each agent as: symptomatic treatment for cognition, symptomatic treatment for behavior, disease-modifying small molecule, disease-modifying immunotherapy, therapeutic device, and stem cells. The category of disease-modifying therapy with small molecules was further divided into amyloid-beta (Aβ) protein approaches, tau-related treatments, and neuroprotective strategies.

Mechanism of action was determined by published data on the compound. Some compounds have more than one activity and were categorized based on what the literature suggests is the primary mode of action.

Data were analyzed for the decade from 2002 through 2012. Registration on clinicaltrials.gov was not mandated until 2007, and participation was greatly increased in 2005 by the decision of the ICMJE to require registration for publication. Data prior to 2007 may be incomplete. In addition, some Phase 1 studies are conducted outside the United States and may not be registered on clinicaltrials.gov. The 2005 decision by the ICMJE and the 2007 decision by the FDA caused, somewhat artificially, increases in the number of trials registered in those years, because ongoing trials were registered regardless of study-initiation date.

In addition, we conducted an analysis of the currently active AD-treatment pipeline (end date, February 28, 2014). This included all agents that are currently registered as active but not yet recruiting, recruiting, or ongoing but not currently recruiting.

We excluded all trials of currently approved medications aimed at supporting the efficacy of an approved compound. We included trials of currently approved medications if the trial included an unapproved test agent that was being used in combination with an approved agent or the approved agent served as an active comparator.

This is a trend analysis aimed at understanding the characteristics and trajectory of change over time in AD drug development, as well as trends across phases and mechanistic categories of AD candidate therapies. Two-sample *t* tests were used to compare trial durations and sample sizes in Phase 2 and Phase 3.

## Results

Table 1 provides an overview of the total number of trials registered over the decade of 2002 through 2012 on clinicaltrials.gov. The 413 trials include 124 Phase 1 trials, 206 Phase 2 trials, and 83 Phase 3 trials. These 413 trials represent 244 unique compounds, with many compounds having more than one trial and some present in more than one phase. More Phase 2 trials were conducted than any other trial type, and fewer Phase 3 trials. Taking the years since 2007 when registration was required, 157 Phase 2 trials and 54 Phase 3 trials were performed. The total number of trials was highest in 2008 (61) and 2009 (72) and has remained approximately stable (45 to 51) over the past 3 years.

**Table 1:** Overview of Alzheimer’s disease clinical trials from clinicaltrials.gov

**Year registered**	**Phase 1**	**Phase 2**	**Phase 3**	**Total**
2002	0	2	3	5
2003	0	5	7	12
2004	1	9	4	14
2005	4	19	9	32
2006	5	14	6	25
2007	16	22	8	46
2008	25	27	9	61
2009	28	30	14	72
2010	16	24	11	51
2011	15	26	4	45
2012	14	28	8	50
Total	124	206	83	413

The pharmaceutical industry sponsors the vast majority of clinical trials for AD drug development. Seventy-eight percent of trials (322 of 413) were funded exclusively by industry, and an additional eight were funded by combinations of NIH ands industry. NIH accounted for 28 (6.7%) of 413 trials, and other organizations such as academic medical centers accounted for 55 of 413.

The United States remains the single world region responsible for most clinical trials (180 (47%) of 385 trials for which the location was recorded on clinicaltrials.gov.) However, cumulatively, more trials are now conducted in international locations (including the U.S. and non-U.S. or exclusively in non-U.S. sites than in the United States only). The proportion of U.S. and non-U.S. trials has remained approximately stable since 2007.

Table 2 presents the trials according to the mechanism of actions of drugs from 2002 through 2012. The largest number of registered trials has been conducted for symptomatic agents aimed at improving cognition (151 of 413 (36.5%)). The next-largest category is disease-modifying small molecules (145 (35.1%) of 413) followed by disease-modifying immunotherapies (76 (18.4%) of 413). Taken together, disease-modifying agents accounted for 53.5% of all trials since 2002.

**Table 2:** Number of trials for agents with varying mechanisms of action

**Year registered**	**Symptomatic for cognition**	**Symptomatic for behavior**	**Disease-modifying small molecule**	**Disease-modifying immunotherapy**	**Therapeutic device**	**Stem cells**	**Total**
2002	1	3	0	1	0	0	5
2003	5	1	4	0	2	0	12
2004	5	2	6	0	1	0	14
2005	10	3	16	3	0	0	32
2006	12	1	8	4	0	0	25
2007	18	1	17	10	0	0	46
2008	23	1	18	15	4	0	61
2009	31	2	19	17	2	1	72
2010	11	2	23	13	2	0	51
2011	17	2	17	5	3	1	45
2012	18	4	17	8	2	1	50
Total	151	22	145	76	16	3	413
Percent	36.56	5.33	35.11	18.40	3.87	0.73	100

**Figure 1:**
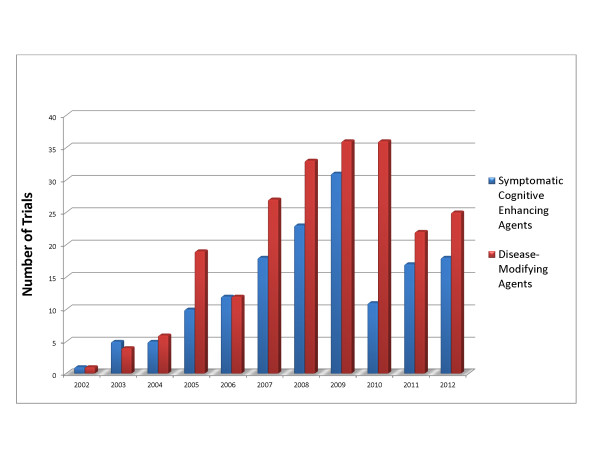
Comparison of number of trials of symptomatic cognitive-enhancing agents and of disease-modifying agents in the 2002 through 2012 period.

Approximately the same number of disease-modifying small molecules have been tested each year since 2007 (17–23). The number of disease-modifying immunotherapies was highest in 2008 through 2010 (13–17) and declined in 2011 and 2012 (5–6). A small number of medical devices and stem cells have entered clinical trials. Figure 1 shows the number of trials of cognitive-enhancing-agent trials and of disease-modifying agents in the 2002 through 2012 period. Table 3 presents the trials for drugs with varying mechanisms of action by trial phase.

**Table 3:** Mechanisms of action of drugs currently in Phase 1, Phase 2, and Phase 3 clinical trials (as of February 27, 2014)

**Unique compounds per MoA (current pipeline 02–27)**	**Phase 1**	**Phase 2**	**Phase 3**	**Total**
Symptomatic for cognition	5	10	10	25
Symptomatic for behavior	2	3	4	9
Disease-modifying small molecule (amyloid-related)	4	5	1	10
Disease-modifying small molecule (tau-related)	3	0	1	4
Disease-modifying small molecule (neuroprotector)	2	19	4	25
Disease-modifying immunotherapy	4	8	3	15
Therapeutic device	2	4	0	6
Stem cells	0	1	0	1
Total	22	50	23	95

Anti-Aβ therapies have dominated AD clinical trials, with 70 of 146 (combined small molecules and immunotherapies) compounds being directed against Aβ compared with 13 compounds addressing tau-related mechanisms and 62 compounds assessing neuroprotective approaches.

The duration of planned treatment exposure in a trial varies by the mechanism of the test agent. In Phase 2, the mean length of trial was 20.0 weeks for symptomatic cognitive-enhancing agents, 16.8 weeks for symptomatic behavior agents, 40 weeks for disease-modifying small molecules (*P* < 0.0001 compared with cognitive enhancer), 61.7 weeks for immunotherapies (*P* < 0.0001 compared with cognitive enhancer), 7.5 weeks for devices, and 10.8 weeks for stem cells.

The mean length of trials in Phase 3 was 34.6 weeks for symptomatic cognitive-enhancing agents, 21.0 weeks for symptomatic behavior agents, 62.1 weeks for disease-modifying small molecules (*P* < 0.0001 compared with cognitive enhancer), 139 weeks for immunotherapies (*P* < 0.0025 compared with cognitive enhancer), and 78 weeks for devices. In all categories, Phase 2 trials were shorter than Phase 3 trials.

The number of patients required for trials is larger for disease-modifying compounds than for symptomatic agents, especially in Phase 3. In Phase 2, the mean number of patients was 199.7 for symptomatic cognitive-enhancing agents, 119.5 for symptomatic behavior agents, 162.61 for disease-modifying small molecules (*P* = 0.28 compared with cognitive enhancer), 102 for immunotherapies (*P* < 0.001 compared with cognitive enhancer), 32.13 for devices, and 20 for stem cells.

The mean number or patients included in Phase 3 trials was 313.8 for symptomatic cognitive-enhancing agents, 215.3 for symptomatic behavior agents, 1,086.0 for disease-modifying small molecules (*P* < 0.0001 compared with cognitive enhancer), 1,321.9 for immunotherapies (*P* = 0.068 compared with cognitive enhancer), and 178.5 for devices. In all categories, fewer patients were included in Phase 2 than in Phase 3 trials.

We examined the progression of compounds from Phase 1 to Phase 2 and from Phase 2 to Phase 3. We reviewed the percentage of compounds that appeared in Phase 1 and were advanced to Phase 2 and the percentage that were listed in Phase 2 and then advanced to Phase 3. Twenty-one compounds that were registered in Phase 1 during the 2002 through 2012 period were also tested in Phase 2 (28% advance rate; 72% attrition rate). Fourteen compounds registered in Phase 2 were advanced to Phase 3 and tested during the decade reviewed (8% advance rate; 92% attrition rate). During the decade reviewed, one compound (memantine) was advanced from Phase 3 to the FDA for review and approval, and 54 compounds were tested in Phase 3 during this period (1.8% advance rate; 98.2% attrition rate; for this calculation, we excluded all current Phase 3 compounds because they may succeed in advancing to the FDA for review). Overall, 244 compounds were assessed in the decade of 2002 through 2012 and one was approved for marketing; excluding the 14 compounds currently in Phase 3, the success rate for advancing agents for regulatory approval is 0.4% (99.6% attrition).

We examined the currently active pipeline of AD therapies to understand the characteristics of agents currently in development (end date as February 28, 2014). Of these, currently 110 trials of AD therapies are ongoing: 26 Phase 1 trials representing 22 unique therapies; 54 Phase 2 trials assessing 49 unique treatments; and 30 trials Phase 3 testing 23 therapeutic compounds. Phase 3 includes six cognitive-enhancing agents, four drugs aimed at improving behavioral symptoms, seven disease-modifying small molecules, one trial of insulin, and three disease-modifying immunotherapies.

## Discussion

This study used the publically available clinicaltrials.gov database to assess the historic trends of AD drug development and to put the current pipeline of agents in perspective. The results demonstrate that detailed interrogation of clinicaltrials.gov can provide insight into longitudinal trends in drug development. A comprehensive database of all clinical trials registered in clinicaltrials.gov, the Aggregate of ClinicalTrials.gov (AACT), has become available [12] and may facilitate further analyses.

In the decade of 2002 through 2012, 244 compounds were assessed in 413 trials for AD. Of the agents advanced to Phase 3 (and excluding those currently in Phase 3), one was advanced to the FDA and approved for marketing (1.8%). Excluding the 14 compounds currently in Phase 3, the overall success rate for approval is 0.4% (99.6% attrition). This is among the lowest for any therapeutic area [13,14].

The developmental time lines for conducting two Phase 3 trials needed to satisfy FDA requirements is substantially shorter for symptomatic agents than for disease-modifying compounds. For symptomatic cognitive enhancers in Phases 2 and 3, the trials were 20 and 34.6 weeks in duration, whereas trials of disease-modifying agents were 47.6 and 90.9 weeks in Phase 2 and Phase 3, respectively. The total duration of a trial is the length of time devoted to recruitment plus the treatment period; in some cases, the trial length included an open-label extension. The period of recruitment varies and is often longer than anticipated by the sponsor, because recruitment of AD patients is slower than expected for many trials [15]. The total time that a compound resides in any phase of the development pathway is a combination of duration of all the trials performed (some may be done concurrently) and time for analysis and decision making.

Progression through the pipeline is not necessarily sequential. Not all compounds tested in Phase 2 or 3 would have been assessed in previous stages. For example, a repurposed compound could be tested in Phase 3, based on data generated in populations with other indications, without necessarily be assessed in Phase 1 or in Phase 2 for AD. Rosiglitazone is an example of such a compound; it was tested in Phases 1, 2, and 3 for diabetes and in Phase 3 for AD. A repurposed compound entering the pipeline in Phase 3 might require testing in Phase 1 (for example, drug-drug interactions studies with antidementia agents in healthy volunteers). Dimebon is an example of this reverse sequencing; this agent had simultaneous Phase 1 trials assessing drug-drug interactions and Phase 3 trials for efficacy.

As drugs progress through the development pipeline, trials become longer and larger; this is especially evident in the programs for disease-modifying compounds. The mean duration of trials in Phase 2 is 47.6 weeks, and the mean duration of Phase 3 is 90.9 weeks. The average number of patients in Phase 2 is 142, and the average number in Phase is 833. The resource requirements for developing disease-modifying agents are greater than those required for symptomatic agents. Phase 2 has been substantially smaller and shorter than Phase 3 for most agents. More-robust Phase 2 programs with better understanding of the molecule might contribute to improving the success rate in Phase 3.

The attrition rate for AD treatment is high, with 72% of agents failing in Phase 1, 92% failing in Phase 2, and 98% failing in Phase 3 in the period observed. If these rates are applied to the current pipeline, 6.4 of the agents in Phase 1 and 4.7 of the agents in Phase 2 will be advanced to the next stage. Of the 14 drugs currently in Phase 3, the data predict that only a very limited chance exists of any being advanced for regulatory review. Predictions of this type will remain conservative until a breakthrough first-in-class agent recalibrates the expectations.

The one agent approved during the decade reviewed (memantine) is a symptomatic cognitive enhancer. Cognitive-enhancing agents are an active area of investigation with 151 of 413 trials in the 2002 through 2012 period devoted to this class of agents.

Two-hundred twenty-one agents have been assessed for disease-modifying potential, and none has shown a drug-placebo difference in favor of active treatment on primary outcomes, although a few agents (seven) are in on-going trials of this class of agent, and their outcome has yet to be determined. Failures in trials may be based on lack of efficacy, excessive side effects, or challenges in trial execution. Trial-conduct failure is suggested by a lack of decline in the placebo group, no effect in an active-treatment comparator arm of the study, or excessive measurement variability. The reasons for trial failures suggest means of enhancing the success of trials, including improved rating strategies, enhanced training, and better patient-selection approaches [16,17]. New means of predicting drug toxicity may reduce the attrition rate attributable to lack of safety [18,19].

Reasons for lack of efficacy in well-conducted trials must also be interrogated to improve the success rate for AD drug development. It has been suggested that use of antiamyloid agents may be optimized by intervening earlier in the disease process before nonamyloid processes prevail and neurodegeneration begins [20-23]. Identifying new disease pathways more amenable to pharmacologic manipulation, improved understanding of the complex neurobiology of AD, and use of combinations of therapies may provide new approaches to AD therapy [24-26].

Most disease-modifying trials have some form of Aβ protein as the pharmacologic target (that is, four of six current Phase 3 compounds of disease-modifying agents target the amyloid-beta protein). One-hundred forty-five (65.6%) of 221 trials of disease-modifying agents registered in the 2002 through 2012 period were directed at this target. The target is unvalidated, and no class of agents has shown efficacy for this target in human clinical trials. Many animal models of amyloidosis have shown biological and behavioral benefit from anti-Aβ agents, creating a “translational gap” between human and animal studies [27-30]. Development of animal models more predictive of success in human trials, diversification of targets within AD, use of rational combinations to address multiple disease pathways simultaneously, and optimizing the selection of patients more likely to be responsive to antiamyloid therapies may all enhance success in AD drug development.

The current AD pipeline is relatively modest, given the enormous challenge posed by this disease. AD is more expensive to the U.S. economy than cardiovascular disease or cancer [31]. Currently, 108 clinical trials for AD therapies are being conducted. This compares with 1,438 ongoing trials for oncology agents. The success rate of development of oncology compounds is 19% [32], encouraging biotechnology and pharmaceutical companies to invest time, effort, and funds in oncology drug testing. Similar successes are needed to spur AD drug development.

The high rate of attrition of compounds requires a constant supply of new approaches (new chemical entities, immunotherapies, repurposed drugs, devices) that can be assessed for efficacy in AD. The pipeline is dependent on a complex drug-development ecosystem of academic laboratories, federal funding agencies, biotechnology companies, venture capital, philanthropy, trial sites, contract research organizations, pharmaceutical companies, advocacy groups, and regulatory agencies. This ecosystem must be supported, grown, and coordinated to improve the success of AD trials and development of desperately needed new AD therapies.

## Conclusion

ClinicalTrials.gov provides a remarkable resource of information regarding drug development for AD and other disorders. Trends in AD drug development over time can be seen, and the movement of the drugs through the pipeline can be monitored. ClinicalTrials.gov has provided comprehensive information since 2007, when registration of clinical trials was required by the FDA. The analyses demonstrate that the number of clinical trials has been declining since the 2008 through 2009 period. The pharmaceutical industry sponsors most drug development for AD, whereas NIH accounts for a relatively small percentage of drug development. The United States has the largest number of clinical trials of any single country, but more clinical trials are conducted outside of the United States than inside of the United States.

Most trials address symptomatic agents intended to improve cognition, but disease-modifying small molecules and disease-modifying immunotherapies are also represented in the drug-development pipeline. More therapies address amyloid-beta targets than any other single target. Phase 2 trials are smaller and shorter than Phase 3 trials, and sponsors have relatively limited experience with most molecules when they enter Phase 3.

Most drugs entering the AD drug-development pipeline have failed; only one agent has been approved since 2004 (memantine). The failure rate since 2002 (excluding agents currently in Phase 3) is 99.6%. Currently, 108 trials of AD therapies represent 94 unique agents. This is a relatively small number of test compounds. The small number of agents in Phase 1 (22) is particularly concerning, as it suggests that relatively few drugs are entering the AD drug-development process. Repurposed agents may enter the pipeline at later phases, but it is unlikely that a large number of repurposed agents will be assessed. The AD drug-development pipeline is relatively small, and the rate of success of AD clinical trials is limited. An urgent need exists to increase the number of agents entering the pipeline and progressing successfully toward new therapy for patients with AD.

## Abbreviations

AD: Alzheimer’s disease; Aß: amyloid beta protein; FDA: Federal Drug Administration; ICMJE: International Committee of Medical Journal Editors; MCI: mild cognitive impairment; MMSE: Mini-Mental State Examination; NIH: National Institutes of Health; NMDA: *N*-methyl-D-aspartate; U.S.: United States.

## Competing interests

Dr. Cummings has provided consultation to Acadia, ADAMAS, Avanir, Boehinger-Ingleheim, Bristol Myers Squibb, Eisai, EnVivo, Genentech, General Electric Healthcare, GSK, Lilly, Lundbeck, Medavante, Merck, Novartis, Otsuka, Pfizer, Prana, QR Pharma, Resverlogix, Servier, Sonexa, Suven, Takeda, and Toyama pharmaceutical companies. TM and KZ have no disclosures.

Dr. Kate Zhong declares that she has no competing interests.

Travis Mortsorf declares that he has no competing interests.

## Authors’ contributions

JLC conceived the project, participated in analyses of the database, reviewed the database for accuracy, reviewed the statistical calculations, drafted the manuscript, and approved the final manuscript. TM constructed the database, participated in analyses of the database, revised the manuscript, and approved the final manuscript. KZ reviewed the database, participated in analyses of the manuscript, participated in revising the manuscript, led the statistical analyses, and approved the final manuscript.
